# Human leucocytes processed by fast-rate inertial microfluidics retain conventional functional characteristics

**DOI:** 10.1098/rsif.2023.0572

**Published:** 2024-03-06

**Authors:** Tom Carvell, Paul Burgoyne, Laura Milne, John D. M. Campbell, Alasdair R. Fraser, Helen Bridle

**Affiliations:** ^1^ Institute of Biological Chemistry, Biophysics and Bioengineering, School of Engineering and Physical Sciences, Heriot-Watt University, Heriot-Watt Research Park, Edinburgh EH14 4AS, UK; ^2^ Tissues, Cells and Advanced Therapeutics, Jack Copland Centre, Scottish National Blood Transfusion Service, Research Avenue North, Heriot-Watt Research Park, Edinburgh EH14 4BE, UK

**Keywords:** cell therapy, microfluidics, inertial focusing, medium exchange, viability, advanced therapy medicinal products

## Abstract

The manufacturing of clinical cellular therapies is a complex process frequently requiring manipulation of cells, exchange of buffers and volume reduction. Current manufacturing processes rely on either low throughput open centrifugation-based devices, or expensive closed-process alternatives. Inertial focusing (IF) microfluidic devices offer the potential for high-throughput, inexpensive equipment which can be integrated into a closed system, but to date no IF devices have been approved for use in cell therapy manufacturing, and there is limited evidence for the effects that IF processing has on human cells. The IF device described in this study was designed to simultaneously separate leucocytes, perform buffer exchange and provide a volume reduction to the cell suspension, using high flow rates with high Reynolds numbers. The performance and effects of the IF device were characterized using peripheral blood mononuclear cells and isolated monocytes. Post-processing cell effects were investigated using multi-parameter flow cytometry to track cell viability, functional changes and fate. The IF device was highly efficient at separating CD14+ monocytes (approx. 97% to one outlet, approx. 60% buffer exchange, 15 ml min^−1^) and leucocyte processing was well tolerated with no significant differences in downstream viability, immunophenotype or metabolic activity when compared with leucocytes processed with conventional processing techniques. This detailed approach provides robust evidence that IF devices could offer significant benefits to clinical cell therapy manufacture.

## 1. Introduction

Since their introduction, cell-based therapies for clinical use have had profoundly positive impacts on patient outcome [1]. All cell therapies require licencing through an appropriate regulatory authority (such as FDA, MHRA or EMA) prior to clinical trial and are described as advanced therapy medicinal products (ATMPs). Hundreds of active clinical trials involving cell therapies are registered with clinicaltrials.gov but currently only 24 therapies have received full market authorization by the EMA to date [2]. The cell types used have primarily been blood leucocytes such as T cells, monocytes and macrophages [3]. In products involving the latter two, the manipulation of both monocyte and macrophage cellular material is of critical importance to ensure the production of ATMPs that comply with regulator established good manufacturing practice (GMP) guidelines. Despite more recent advances in cellular therapy manufacturing, there remain sizeable challenges and a need for new or improved technologies to provide processes that are consistent, cost-effective and scalable [4].

These technologies are required because cellular therapies differ from traditional medicinal products as they contain living cells that require precise operator manipulation and processing steps such as *ex vivo* culture [5]. Great care is needed throughout the manufacturing procedure to ensure the cells are maintained within optimum parameters [6], and that the final drug product meets the required ATMP specifications prior to administration to the patient [4].

Cell therapy manufacturing frequently uses centrifugation, filtration or label-based separation techniques to transfer cells between different buffers and media and for the final formulation of the drug product into a suitable excipient. There are a number of traditional and emerging technologies suitable for cell therapy manufacturing and which are capable of cell manipulation, buffer exchange and volume reduction and these have been reviewed elsewhere [7–9]. However, the widespread adoption of a label- and centrifugation-free, cost-effective and high throughput system has yet to be realized and many research and development and contract development and manufacturing organizations (CDMO) continue to use a variety of processing methodologies. Table 1 shows a list of relevant technologies capable of cell manipulation and medium exchange in cellular therapy manufacturing.

**Table 1.** Methods of cell manipulation and buffer exchange with applications in cellular therapy manufacturing.

processing method	centrifugation^a^	spinning membrane filtration (Lovo)^a^	inertial focusing system	CliniMACS prodigy^b^
application	cell concentration (volume reduction), medium/buffer exchange	cell concentration (volume reduction), medium/buffer exchange	cell concentration (volume reduction), medium/buffer exchange, cell sorting	cell concentration (volume reduction), medium exchange, cell depletion/enrichment, cell expansion/culturing
throughput	low	high	high	high
open or closed process	open	closed	closed	closed
processing time	protocol/cell type dependent (17–90 min)	protocol/cell type dependent (17–90 min^−1^)	cell type and application dependent. Capable of 10 ml min^−1^	variable (defined by process)
number of processing steps	variable (minimum 1, often 3–6)	variable (minimum 1, often 2–6)	one, repeated if larger volume reduction or greater medium exchange required	variable (defined by process)
volume reduction achievable	operator dependent, final volume achievable <1 ml	variable, final volume approximately 50 ml	up to 75% of volume reduction of inlet volume per processing step. Minimum final volume approximately 2–5 ml	variable, but up to four product bags (30–100 ml) with one QC bag (10 ml)
cost	moderate upfront, small consumables cost	large upfront, large consumables cost	moderate upfront, moderate consumables cost	large upfront, large consumables cost

^a^Processing times and number of steps vary. Data obtained from [10].

^b^Data obtained from [11].

Note: centrifugation column includes data from studies using manual centrifugation, CellSaver 5+ and COBE 2991.

As an alternative to traditional centrifugation, spinning membrane filter devices such as the Lovo (Scale Ready/Fresenius) can be used to process cells, separating intact cells from debris and platelets, as well as for volume reduction. A multi-centre study of Lovo processing of a variety of cell types found that cell viability (Lovo: 80%, manual: 70.3%) and recovery (Lovo: 82.3%, manual: 76.7%) was comparable to manual approach processing using defined volume reduction protocols [10]. However, despite these improvements, average processing times increased by nearly 40% compared with manual cell processing [10] and costs were significantly higher due to the need for dedicated tubing sets. A single-centre study reported that both CD34+ haematopoietic stem cells and mononuclear cells processed using the Lovo met internal study requirements (greater than 50% cell recovery) and the authors indicated that despite an overall longer procedure, the Lovo offers substantial advantages relative to processing manually or with the COBE 2991 [12]. These studies focus primarily on recovery and cell viability, but little is known about any impacts on cell health or other longer-term effects. It has been reported that ultra-scaled-down processes involving pelletization and resuspension of cells (i.e. centrifugation) cause damage to both cell membrane integrity and cell surface proteins [13,14] which could explain higher cell viability and recovery reported in studies using the Lovo.

Microfluidic devices are an attractive option which potentially address key issues in cell therapy manufacturing due to their ability to precisely manipulate the spatiotemporal behaviour of fluid microenvironments in which the cells reside [15]. Microfluidic devices contain microchannels that are ideal for cell therapy manufacturing as they are well suited to the size of human cells, while offering the potential for automation and scalability alongside a closed system designed to reduce the possibility of operator error and to increase processing repeatability [16]. Furthermore, microfluidic devices are highly flexible in their design and can be integrated with other components to fulfil a range of functions required for biological processes [17].

The microfluidic device used in this study has a spiral microchannel design with a rectangular cross-section and exploits both inertial focusing (IF) and Dean flow; the physics of these forces have been extensively reviewed [17–20]. Briefly, IF is a phenomenon whereby randomly distributed, suspended particles (electronic supplementary material, figure S1*a*) migrate across streamlines to well-defined positions within the cross-section of a flow [21,22]. IF arises from a balance between hydrodynamic forces within fluid flow generated as a result of the microchannel geometry, a fluid Reynolds number (greater than 1) and various other parameters, including the shape, dimensions and deformability of the cells [23]. The result of IF is that suspended particles or cells reach an equilibrium state on focal positions at the cross-section of the channel (electronic supplementary material, figure S1*b*). Differences in the parabolic velocity profile across the cross-section of the fluid, in conjunction with microchannel curvature, generates centrifugal forces that are directed radially outward at the microchannel cross-section. To ensure mass conservation, there are outward flows at the centre of the channel and inward flows at the upper and lower walls [24]. These forces also generate a recirculation of fluid orthogonal to the direction of flow, causing Dean flow (electronic supplementary material, figure S1*c*), whereby cells experience a transverse drag force [25]. Cell focal equilibrium positions are mediated through Dean flow and final positions are highly dependent on flow and cell characteristics. The IF prototype in this paper exploits the use of a sheath flow (fresh culture medium) whereby minimal mixing of fluids in the microchannel occurs, but secondary Dean flow causes a recirculation of the fluid so that cell focal positions exit at the outlets predominantly within the fresh medium [26].

The use of IF and Dean flow as an efficient separator of particles and cells in a variety of devices has been previously reported with various device architectures [20,26–31]. However, these devices are yet to be employed as tools within cell therapy manufacturing.

Shear forces in microfluidic devices arise due to the velocity gradient within the flow and the force is exerted on the cells within the fluid. Although high shear forces have been reported to impact cellular viability [32], there is little research on cellular metabolomics post-processing. Assessment of devices for use in cell separation requires measurement of key parameters such as viability and recovery to determine device performance and effects of processing on cellular material [33–40]. For devices capable of buffer exchange, studies often cite trypan blue dye or dimethyl sulfoxide removal (from processing cryopreserved material) as a key marker for device efficiency or use particles to determine recirculation patterns [10,26,41–43]. Though these parameters are important, they are relatively simplistic determinants and do not examine potential downstream effects on cell function, which is relevant in cell therapies where source material is differentiated into a final product (such as monocytes to macrophages or dendritic cells).

In this paper, we outline the development and testing of a label-, centrifuge- and filtration-free microfluidic prototype device capable of concurrent cell manipulation and buffer exchange. In particular, this IF device operates at much higher throughput of cell processing than many other IF devices [20] and the study focuses on assessing the quality, viability and fate of human leucocytes processed using this device as there is little published data on cellular effects of processing. The cell analysis uses multi-parameter flow cytometry, microscopy and evaluates post-processing changes in cellular metabolism using gas chromatography.

## 2. Methodology

### 2.1. Device design and fabrication

**Figure 1.**
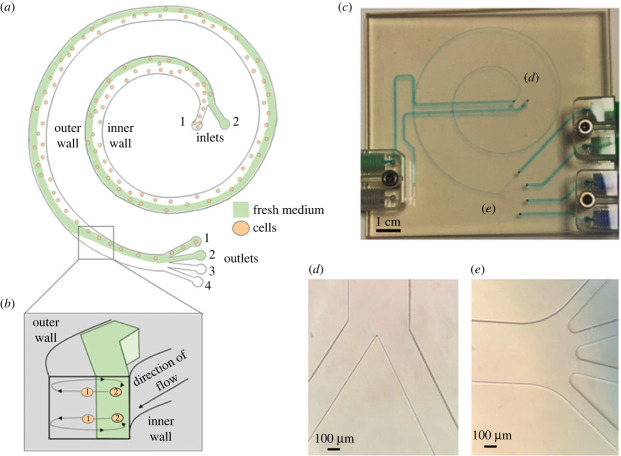
Inertial focusing microfluidic device for cell separation, medium exchange and volume reduction. (*a*) Schematic depicting the injection of cells (orange circles) at inlet 1 and fresh medium (green) at inlet 2 before inertial focusing-driven cell sorting in conjunction with a Dean-flow-mediated medium exchange mechanism upstream of the outlets (numbered 1–4). (*b*) Formation of Dean vortices (curved arrows) causes a recirculation of fluid orthogonal to the direction of flow and a disruption to stable equilibrium positions (1) of inertial-focused particles and the formation of new off-centre equilibrium positions (2) within the flow of fresh medium fluid. (*c*) An image of the 2-inlet, 4-outlet inertial-focusing microfluidic device primed with blue dye for visualization of the microchannel, (*d*) inlets and (*e*) outlets.

For the experiments described, figure 1*a* illustrates the schematic operation of the device. The design includes a 1.75-loop spiral microchannel with rectangular cross-section (W: 480 µm, H: 80 µm, L: 220 mm) (figure 1*b*), and 13.8 mm spacing between loops (figure 1*c*). The microfluidic device was manufactured (Epigem Ltd, GB) using polymethyl methacrylate and has two inlets (figure 1*d*) of equal size (W: 240 µm, H: 80 µm), and four outlets (figure 1*e*) of equal size (W: 120 µm, H: 80 µm).

**Figure 2.**
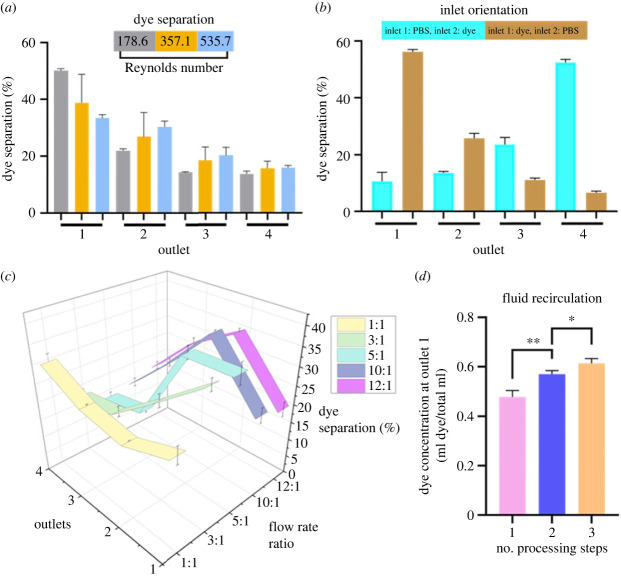
Dye exchange with PBS as a modelling tool for medium exchange*.* (*a*) Efficiency of dye separation. Processing was performed at different flow rates (characterized by Reynolds number) with an inlet flow rate ratio of PBS 3 : 1 dye. Dye was injected at inlet 1 and PBS was injected at inlet 2. Mean ± s.d.; *N* = 3. (*b*) Efficiency of dye exchange following processing with different starting fluid inlet orientations. Processing was performed at total flow rate of 3 ml min^−1^ (Re = 178.6) with an inlet flow rate ratio of PBS 3 : 1 dye. Mean ± s.d.; *N* = 5. (*c*) Efficiency of dye exchange following processing at 9 ml min^−1^ (Re = 535.7) with various inlet flow rate ratios between PBS and dye. Mean ± .s.d.; *N* = 3. (*d*) Dye recovery at outlet 1 as a percentage of initial dye concentration following processing. Sample collected from outlet 1 was recirculated for the next processing step. See figure 1 for inlet and outlet numbering. *p < 0.05, **p < 0.01. Mean ± s.d.; *N* = 3.

Predicting the behaviour of cells within IF devices is challenging, with authors relying on trial and error and device reiteration; however, various design rules have been proposed and guided our design process in selecting the device dimensions. The microchannel height was chosen to satisfy the following criterion [44,45]:

$$\frac{a_{p}}{0.5}\leq h\leq \frac{a_{p}}{0.07},$$

where *a_p_* is cell (or particle) diameter and *h* is the microchannel height. The microchannel width was chosen to fulfil an aspect ratio of 6, which was reported as optimal for faster processing rates for a similar design [46]. A 1.75 loop spiral design was chosen to replicate optimal buffer exchange reported previously [26]. The equally sized inlet channel design allows for fluids to be introduced into the device in either orientation. The geometry of the outlets was designed following preliminary testing with an earlier iteration of the IF device, and the revised prototype discussed in this publication reflects that testing to allow for outlets aligned with cell focusing positions.

### 2.2. Capacity for medium exchange

Blue food dye (Dr Oetker, Germany) was diluted to 1 in 10 with phosphate-buffered saline (PBS; Gibco Ltd, UK) and co-injected alongside standard PBS at the inlets of the IF device at different flow rates and fluid collected at the outlets. Outlet samples were analysed by colorimetry using a plate reader (MultiSkan, ThermoFisher Scientific, UK) to determine the concentration of dye collected at each outlet at different flow rates.

### 2.3. Cell preparation and culture

Buffy coats (enriched leucocyte fraction) were obtained from blood collected from healthy donors by the Scottish National Blood Transfusion Service (SNBTS) under appropriate Sample Governance management (SG 19 approx. 27). Peripheral blood mononuclear cells (PBMC) were isolated by density centrifugation using LeucoSep tubes (Greiner Bio-One, UK) containing Ficoll (Merck, Germany) as per standard, in-house SNBTS protocols. The PBMC were resuspended in RPMI 1640 medium (Gibco), supplemented with 5% AB serum and 100 U ml^−1^ penicillin/streptomycin (pen/strep) (Gibco) and diluted to 3 × 10^6^ cells ml^−1^ for processing. Where applicable, PBMC were cultured overnight in the same medium at 37°C, 5% CO_2_ and harvested using trypsin-mediated enzymatic disaggregation (TrypLE, Gibco) for downstream analysis.

Monocytes were isolated from PBMC using magnetic bead selection using anti-CD14 microbeads (Miltenyi Biotec, UK) as per an established GMP-compliant protocol [47]. Briefly, anti-CD14 microbeads were mixed with PBMC and incubated at 4°C for 20 min. After washing in PBS supplemented with EDTA (Gibco) and human serum albumin solution (Alburex, CSL Behring; PEA buffer), monocytes were isolated by positive selection using a magnetic column and diluted to 3 × 10^6^ cells ml^−1^ for processing.

### 2.4. Device operation

The microfluidic device was first sterilized with 70% isopropyl alcohol (IPA), then washed and primed using sterile RPMI 1640 medium. For experiments requiring post-processing cell culture, the device was set up within a microbiological safety cabinet (MSC) to maintain sterility. The PBMC suspension was injected into the microfluidic device at inlet 1 (figure 1*a*) and cell-free medium was injected at inlet 2 using a neMESYS 1000 N (Cetoni GmbH, Germany) syringe pump, controlled using Cetoni neMESYS UI software. The processed cell material was collected from all outlets.

IF processing requires laminar flow conditions and the avoidance of turbulent flow, which together require satisfying 1 < Reynolds number (Re) > 2000–2600 [48]. With these parameters, predicting flow patterns and cell behaviour in IF devices is challenging and so a range of flow conditions were investigated. Buffer flow rates were tested between 1 and 15 ml min^−1^ and correspond to the Re, cell residency time within the microchannel and the shear stress applied to the cells as listed in table 2.

**Table 2.** Inlet pressure, calculated dyne cm^−^^2^, flow rates and corresponding Re used in experiments with the microfluidic device.

inlet pressure (PSI)	40.6	136.3	214.7	258.2	388.7	440.9	680.2
dyne cm^−2^	2 799 271	9 397 554	14 803 043	17 802 263	26 799 921	30 398 984	46 898 139
flow rate (ml min^−1^)	1	3	5	6	9	10	15
Re	59.5	178.6	297.6	357.1	535.7	595.2	892.9
cell residency time within microchannel (s)	0.51	0.17	0.10	0.08	0.06	0.05	0.03

### 2.5. Analysis of cell viability

All newly isolated PBMC were analysed before and after processing to determine baseline cell health characteristics and cell count. To determine viability, 10^6^ cells were incubated in RPMI with the viability stain DRAQ7 (ThermoFisher Scientific, UK) as per manufacturer's instructions and analysed using a MACSQuant MQ10 flow cytometer (Miltenyi Biotec, Ltd). Apoptosis was quantified by incubating 10^6^ cells in RPMI with CellEvent Caspase-3/7 Green Flow Cytometry reagent (Invitrogen, UK) at 37°C for 30 min. The PBMC were subsequently washed and stained with DRAQ7, as previously described, before analysis by flow cytometry. PBMC were gated to exclude doublets and debris, and the single-cell population was then analysed to quantify the dead (DRAQ7+) or apoptotic (CellEvent+) cells.

### 2.6. Differentiation of monocytes after microfluidic processing

Monocytes were isolated as described earlier and processed at 3 × 10^6^ cells ml^−1^ with the IF device as described in §2.3. Benchtop (unprocessed) and centrifuged (current standard) control monocyte samples were also generated. Processed monocytes were transferred to a six-well cell culture plate containing TexMACS medium and macrophage colony-stimulating factor (100 ng ml^−1^) and incubated at 37°C, 5% CO_2_ for 5 days to generate macrophages. After 5 days, the culture medium was replaced with TexMACS with IFN-γ (50 ng ml^−1^) and lipopolysaccharide (50 ng ml^−1^) for M1 polarization of macrophages, or TexMACS with IL-4 (20 ng ml^−1^) for M2 polarization of macrophages. Cells undergoing macrophage polarization were incubated at 37°C, 5% CO_2_ for 48 h prior to analysis.

### 2.7. Analysis of cell function and phenotype by flow cytometry

At all timepoints for analysis, cells were washed with PBS, and detached using TrypLE as previously described. The trypsinization reaction was quenched using excess TexMACS medium, and the cells were resuspended in PEA before incubation with a cocktail of fluorophore-conjugated antibodies (electronic supplementary material, table S1) for 30 min at 4°C. The cells were then washed to remove excess antibody, resuspended in PEA and stained with DRAQ7 as previously described. Data were acquired using an LSRFortessa flow cytometer (BD, USA), and all data files were analysed using FlowJo software version 7 (Treestar Inc.).

### 2.8. Analysis of metabolic changes post-processing

To assess changes in cell metabolism post-processing, spent culture medium was analysed by gas chromatography. Post-processed cells were cultured as described previously. Centrifuged cells (300*g*, 5 min) and unprocessed cells were also cultured as controls. After 24 h, the medium was collected and spun down to remove any cells. Media samples from post-processed monocytes were also taken after 24 h, 5 days of macrophage differentiation culture and 48 h after macrophage polarization. Media samples were frozen until use and thawed immediately prior to analysis. Samples were tested for pH, glucose content and lactate accumulation by gas chromatography (RAPIDPoint500, Siemens), using fresh RPMI as a baseline for metabolites.

### 2.9. Statistical analysis

Statistical analyses were performed using IBM SPSS Statistics 28 software (IBM, USA) and Prism 9 (Graphpad 9, USA). All statistical tests are one-way ANOVA (with Bonferroni correction) unless otherwise indicated. Statistical significance was assumed at *p* < 0.05. Data are presented as mean ± standard deviation (s.d.). *N* refers to biological replicates.

## 3. Results and discussion

The microfluidic device used in this study was tested for efficacy in buffer exchange as this is a primary requirement for transferring cultured cells from medium to a suitable excipient for administration or cryopreservation. The efficiency of the process in this device was assessed using colorimetric analysis to determine changes in dye content as a surrogate measure of medium exchange.

### 3.1. Inertial focusing device for medium exchange

Diluted food dye (1 in 10) was injected into one inlet, alongside PBS at the other inlet, to investigate the capacity of the IF device to perform medium exchange. Outlet samples were analysed using a colorimeter, and the dye was used as a visual marker for medium exchange. In this investigation, a flow rate of 3 ml min^−1^, with a flow rate ratio of PBS 3 : 1 dye, was the optimal processing condition for dye collection to the target outlet 1 (mean 50.1%) as a proportion of total collected dye. As flow rate increased, the dye became more spread across the microchannel cross-section and a greater proportion of the dye was collected at non-target outlets (2–4). The mean percentages of dye collected at outlet 1 were lower when run at higher rates of 6 ml min^−1^ and 9 ml min^−1^ (38.8% (*p* = 0.122) and 33.5% (*p* = 0.031), respectively; figure 2*a*). It was determined that the efficiency of medium exchange was inlet dependent with 4% (*p* < 0.01) more dye collected depending on orientation of fluid injection. If injecting PBS at inlet 1 and dye at inlet 2, 52% of dye was collected at the target outlet 4, whereas if the inlet fluids were reversed, 56% of dye was collected at the target outlet 1 (figure 2*b*). Despite this, the device can achieve above 50% medium exchange with one processing step irrespective of inlet fluid orientation which allows for some flexibility in the final desired processing method.

**Figure 3.**
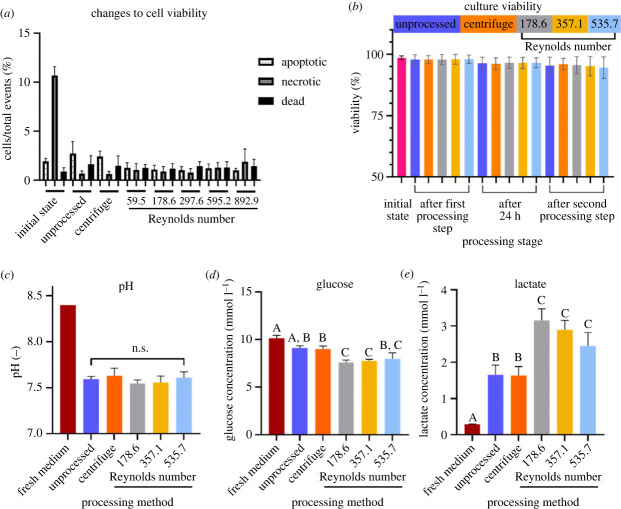
Assessment of inertial focusing (IF) microfluidics device processing effects on PBMC. (*a*) PBMC were processed and immediately analysed by flow cytometry to determine viability. PBMC health (inlet concentration: 3 × 10^6^ cells ml^−1^). Mean ± s.d.; *N* = 3. (*b*) Culture viability of PBMC over time following processing by IF device or centrifugation. PBMC were processed, cultured over 24 h before pre- and post-processing viability tests were performed. Mean ± s.d.; *N* = 3. Inlet concentration: 3 × 10^6^ cells ml^−1^, *N* = 3. (*c*) pH of medium from PBMC cultured for 24 h following processing using centrifugation or by the IF device. Statistical analysis was performed using multivariate one-way ANOVA with Bonferroni correction. Mean ± s.d.; *N* = 3*.* (*d*) Glucose and (*e*) lactate concentrations in the medium of PBMC cultured for 24 h following processing by either centrifugation or the IF device. Statistical analysis was performed using multivariate one-way ANOVA with Bonferroni correction. Groups that do not share a letter are significantly different (*p* < 0.05) from each other. Mean ± s.d.; *N* = 3. Note: corresponding flow rates for each Re are available in table 1.

The use of different flow rate ratios (PBS:dye) at a total flow rate of 9 ml min^−1^ (Re = 535.7) was investigated. Although less dye was collected at outlets 1 and 2 at 3 : 1 flow rate ratio compared to 5 : 1 ratio (3 : 1, 65%; 5 : 1, 72%), more dye was collected in the target outlet 1 at the 3 : 1 ratio (37%) relative to 5 : 1 ratio (36%) although these data did not reach significance (*p* = 0.671). We then investigated if dye exchange could be improved upon further with additional re-circulations of sample collected at outlet 1 (the target outlet that contained the most dye) at a flow rate of 3 ml min^−1^ (PBS:dye, 3 : 1). Dye comprised 47.9% of total sample volume after one circulation and increased to 56.9% (*p* = 0.004) after two circulations and 61.3% (*p* < 0.001) after three circulations (figure 2*d*) demonstrating a small additive effect of multiple re-circulations. Further investigation is required to assist with revised device designs with the aim to perform complete solution exchange.

### 3.2. Effects on peripheral blood mononuclear cells through processing with the inertial focusing device

IF-based devices have been investigated for specific aspects of human leucocyte processing such as detection of cancer cells [49,50]. Given that the cells experience high inlet pressures for short periods of time (table 2), we compared post-processing effects on cell viability following processing using the IF device with the impact of the force applied on cells for longer periods of time as experienced during processes requiring centrifugation. Although there was no evidence of an impact on viability, it has previously reported that the centrifugation of leucocytes affected the fluid shear response and their subsequent reinfusion to rat models resulted in a significant increase in tissue migration relative to non-centrifuged leucocytes [51]. Another platform, the CliniMACS Prodigy is GMP-compliant, uses centrifugation for medium exchange but is automated, with a closed processing system and can perform entire manufacturing procedures within one device. However, this requires expensive consumables and has inherent rate limiting steps for further processing [52]. Critically, it has been demonstrated that the processing of cells with the CliniMACS Prodigy has little impact on viability and can be used with a variety of cell types and for different applications [5].

By contrast, there is little published evidence for the effects of IF processing on leucocyte health and downstream differentiation potential. Although this prototype IF device is not intended for use in mixed cell populations, as an initial assessment of tolerability, we investigated these effects on freshly isolated donor human PBMC to assess the outcomes of microfluidic processing on cell viability and function. PBMC suspended in medium were injected into the IF device alongside culture medium over a range of flow rates. Immediate (less than 1 h) post-processing analysis of cell viability demonstrated no significant differences relative to standard processing (centrifugation) and unprocessed benchtop controls (figure 3*a*). Although it has been previously reported that cells experiencing high shear stress do exhibit subsequent reduced viability [37], data presented here agree with other studies which have shown minimal changes in viability after processing using an IF device though these studies used cell lines which are not representative of human donor PBMC [53] or lower throughput processing rates [9]. To investigate longer-term downstream effects, PBMC were cultured for 24 h following processing and viability remained above 90% for all IF device samples with no significant difference between the methodologies (figure 3*b*). As an indicator of cell function, culture media from these overnight incubations were collected and analysed by gas chromatography to identify any changes in pH or metabolites from culture medium. There was no difference in culture pH between processing methods (figure 3*c*), but cells processed using the IF device (at 5 and 10 ml min^−1^) demonstrated increased glucose usage and lactate production than cells with the other processing techniques (figure 3*d*,*e*). Despite different donor isolates there was little variation in post-processing effects on viability and metabolic activity, although it would be expected that different cell subtypes could behave differently under high processing rates. This suggests the shear forces experienced by the cells during IF device processing caused a transient increase in metabolic activity in some cell types with a consequent upregulation of pathways that mediate glucose use. There is unlikely to be any significant impact on cell health or function as a consequence of this transient metabolic activity.

#### 3.2.1. High-throughput CD14+ monocyte processing

**Figure 4.**
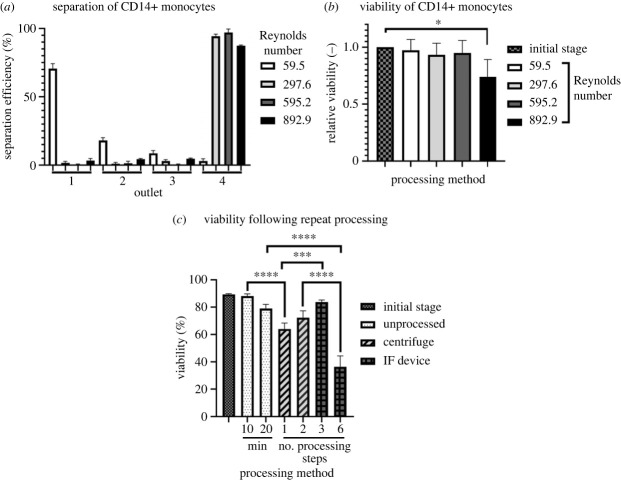
Rapid separation of CD14 + monocytes was well tolerated. (*a*) Efficiency of separation of isolated monocytes at various flow rates. Separation of at least approximately 90% was achieved at 5 ml min^−1^ (Re = 297.6), 10 ml min^−1^ (Re = 595.2) and 15 ml min^−1^ (Re = 892.9). Mean ± s.d.; *N* = 3. See figure 1 for outlet numbering. (*b*) Immediate change in CD14+ human cell viability (inlet conc.: 2 × 10^6^ cells ml^−1^) following processing by the inertial focusing microfluidic device. The viability is relative to the initial viability of unprocessed cell material. Statistical analysis of the data was performed using multivariate one-way ANOVA with Bonferroni correction based on raw cell viability data. Mean ± s.d.; *N* = 3. (*c*) Viability of post-processed CD14+ monocytes by processing method (*n* = 3). IF, inertial focusing. IF device processing at 5 ml min^−1^ (Re = 297.6), *p < 0.05, ***p < 0.001, ****p < 0.0001. Mean ± s.d.; *N* = 3.

A key aspect of IF processing of human material for cell therapies is to determine efficacy in processing a relevant cell type. A major cell therapy manufactured by SNBTS is an autologous macrophage product for treatment of liver cirrhosis [47,54], so processing of the progenitor monocytes was assessed using the IF device. The CD14+ monocytes were isolated from PBMC by magnetic bead isolation to high purity (greater than 90%) and processed with the device, compared with current standard centrifuge-based technique. At the optimal flow rate of 10 ml min^−1^ (Re = 595.2), 97% of CD14+ monocytes were separated to outlet 4, with a slightly reduced separation efficiency of 94% and 87% for processing flow rates of 5 and 15 ml min^−1^, respectively (figure 4*a*). The immediate post-processing viability of CD14+ monocytes showed no significant differences in viability in cells processed at ≤10 ml min^−1^ compared to the controls. When processing through the IF device was increased to a flow rate of 15 ml min^−1^ (Re = 892.9), cell viability decreased to 73% (*p* = 0.004) of the initial viability (figure 4*b*) indicating an upper limit for suitable flow rates. A flow rate of 5 ml min^−1^ (Re = 297.6) was selected for a comparative study to determine the effect of single and multiple processing steps on cell viability (figure 4*c*). The benchtop control sample was maintained at room temperature for the duration of time required for either three or six circulations of cells through the IF device (and equivalent to one or two centrifugations). Though there was a small decrease (4%) in viability after three re-circulations using the IF device at 5 ml min^−1^, this was significantly less than the effects of centrifugation and highlights the potential benefit of IF compared to standard processes. The increased cell death seen after six rounds of IF processing indicates that there is a cumulative effect on the cells and demonstrates an upper limit to the number of times that the cells can tolerate repeated passage through the device.

#### 3.2.2. Post-processing effects on the mononuclear phagocyte system

##### 3.2.2.1. Monocyte differentiation

**Figure 5.**
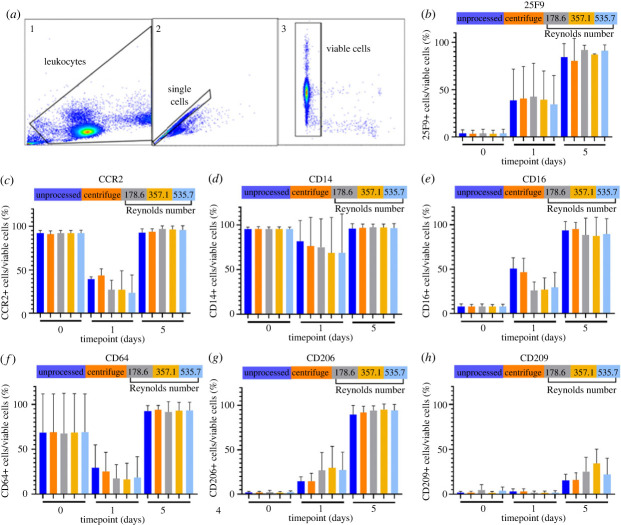
Viability and phenotype of monocytes and macrophages following different processing methods. (*a*) Representative flow cytometry sequential gating used to identify and analyse leucocytes (1) with doublet (2) and dead cell exclusion (3). Viable single cells were then further analysed for cell surface markers (not shown). (*b–h*) Surface marker positivity as a percentage of total viable cells over 5 days following processing. Mean ± s.d.; *N* = 3. Note: the numbers stated as independent variables are the Reynolds number for the flow conditions used during processing with the inertial focusing device. See table 2 for corresponding flow rates.

Macrophages for autologous or allogeneic cell therapy are usually generated from the differentiation of isolated CD14+ monocytes and it is therefore imperative that any changes in downstream cellular phenotype and function resulting from new bioprocessing technology are investigated. To assess this, isolated monocytes were processed through the IF device, cultured with M-CSF, and resulting macrophage cultures were analysed by flow cytometry using sequential gating (figure 5*a*) to assess viability, cell surface marker expression and cellular metabolomics in comparison with standard unprocessed and centrifugation controls.

Over 5 days of monocyte differentiation, there were no significant differences in cell health between processing methods and all samples had viabilities exceeding 80% throughout the experiment (data not shown). These data provide clear evidence that the shear stresses generated by processing through this IF device had minimal effects on longer-term monocyte viability and support previous data from experiments investigating the negligible impact on viability by the IF device processing of PBMC (at flow rates characterized at Re < 600; figure 3).

The analysis of cell surface markers was used to immunophenotype cell populations following bioprocessing. Previous studies have characterized cell lineages by cell phenotype, and the relevant markers have been summarized in electronic supplementary material, table S2. It should be noted that surface marker expression does not necessarily infer function and a wider assessment of function post-processing should be performed. However, these analyses do provide a good insight to viability and phenotype, with single-cell resolution, and have previously been used as a method of quality control for ATMPs [54] in accordance with GMP guidelines issued by the European Medicines Agency and the US Food and Drug Administration [55].

Monocytes can be divided into three sub-populations: classical (CD14+/CD16−), non-classical (CD14 dim/CD16+) and intermediate (CD14+/CD16+) [56]. As a myeloid lineage marker and key protein involved in the regulation of phagocytosis, CD14 expression should remain relatively stable throughout the differentiation process [57]. There were no significant differences in CD14 expression between processing cohorts on any days during the differentiation process (figure 5*d*), although a small decrease was seen in all groups at day 1 which recovered by day 5. This appeared to decrease with increasing flow rates, suggesting that CD14 expression is at least transiently susceptible to shear stress [58]. CD16 expression was initially low (less than 10% of cells), reflecting the small proportion of non-classical or intermediate monocytes in the blood. These data are supported by a study of 16 healthy human donors that reported that approximately 84.8% (±5.6%) of monocytes were characterized as CD14+ CD16− classical monocytes [59]. Expression of CD16 increased over culture to reach peak positivity at day 5 and there were no statistically significant differences between processing cohorts (figure 5*e*). Furthermore, at day 5, most cells across all processing cohorts (mean greater than 85%) were CD16+ with no significant differences in positivity, indicating that the processing method did not impact on CD16 expression. Processing of monocytes during manufacturing is therefore possible using IF devices, with little long-term effect on viability or surface phenotype.

**Figure 6.**
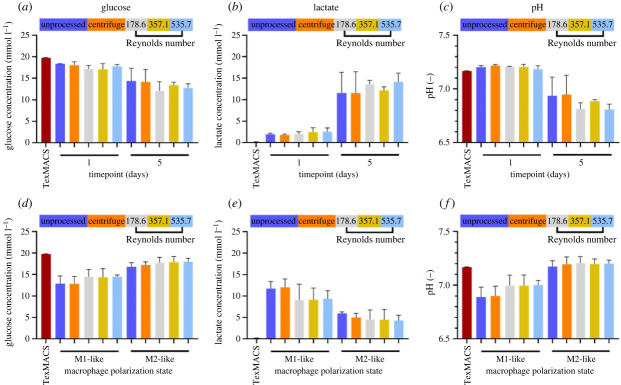
Analysis of culture medium by gas chromatography. (*a–c*) Changes in glucose and lactate concentration, and pH in culture medium over 5 days following monocyte processing. (*d–f*) Changes in glucose and lactate concentration, and pH in culture medium over 2 days following macrophage polarization. Mean ± s.d.; *N* = 3.

The expression of 25F9, a marker of mature macrophages, increased throughout the differentiation of monocytes and a high proportion (greater than 90%, mean value) of the macrophage populations on day 5 were 25F9+ (figure 5*b*) in all cohorts. The observed increase in 25F9 expression over time supports a previous study that reported that exposure to either toll-like receptor agonists or M-CSF stimulates monocyte differentiation towards a 25F9+ macrophage population [60]. The monocytes all expressed low levels of the mannose receptor CD206 (figure 5*g*) at day 0. By day 5, there was strong expression of CD206 with no statistically significant differences between different approaches indicating that the processing did not affect the expression of the receptor on differentiated non-polarized macrophages. CD209 expression followed a similar pattern to CD206 but by day 5 about 40% of macrophages were CD209+. This could be explained by a study that reported CD209 expression in monocytes was IL-4 dependent and IL-4 treated monocytes were negative for CD14 expression, which we did not observe in our analysis of CD14 expression. Additionally, a regression-tree based analysis of a high-dimensional single-cell dataset revealed that CD209 is only constitutively expressed on M2-like polarized macrophages [61]. Taken together these data indicate there was no impact on the capability of processed monocytes to differentiate into non-polarized macrophages as a result of IF device processing, highlighting the suitability of these devices in manufacturing processes.

The antibody Fcgamma receptor I CD64 was expressed by nearly all monocytes on day 0 (median greater than 90%), except for cells derived from one donor that had an unusually low level of expression (approx. 20% of cells). At day 1, CD64 expression was greatly reduced in all cohorts and by day 5 nearly all cells in all processing cohorts were positive for CD64. Both monocytes and macrophages are known to constitutively express CD64 [62] but we observed a reduction in CD64 expression at 24 h following processing in all groups with no statistically significant differences between different processing methods. Like CD14, expression of CD64 may be sensitive to high shear stress. The expression of CCR2, a critical receptor that mediates CCL2-dependent migration for the recruitment of monocytes to an inflammatory site [63], followed a similar pattern to CD64. More than 90% of monocytes expressed CCR2 on day 0 before a large reduction in positivity was observed on day 1 and by day 5, the monocyte-derived macrophage population had returned to greater than 90% of cells expressing CCR2 (figure 5*c*). High shear stress has been linked to monocyte release of CCL2, which in culture could result in the decreased expression of CCR2 seen here through receptor internalization [64]. The high expression of CCR2 at day 5 is unexpected, although does not differ between conditions suggesting this is not linked to processing method. These data show that although shear stress can affect surface marker expression, for CD64 and CCR2 these reductions are transient and recoverable in culture. There were no significant differences between cohorts of cells processed by different methods meaning that cells processed with the IF device would continue with migration, wound-healing and other cellular functions with similar efficacy to cells conventionally processed via centrifugation.

##### 3.2.2.2. Macrophage polarization

Macrophage polarization occurs in response to changes in the microenvironment, such as signalling by various cytokines, and ultimately results in the acquisition of cellular phenotypes that can be broadly defined as pro-inflammatory (M1) or anti-inflammatory (M2), though this is a generalization [65]. It is increasingly apparent that these two classes of polarized macrophage play significant roles in a range of diseases such as cancers [66], liver diseases [67] and autoimmune disorders [68]. This research has helped with the identification of potential applications for macrophage-mediated treatment of these diseases through a variety of mechanisms such as autologous cell therapies [47], macrophage targeting immunotherapies [69] and macrophage metabolic pathway-targeting therapies [70]. Like other cellular therapies, the manufacturing of macrophages relies on conventional technologies for cell manipulation and buffer exchange and would benefit from next-generation bioprocessing protocols. Here, we discuss the post-processing effects over 48 h following exposure to either IFN-γ and LPS or IL-4 to drive an M1-like or M2-like polarization.

The monocyte-derived macrophages (as described in the monocyte differentiation experiment) were then incubated in polarization-inducing culture medium for a further 48 h. At day 0 of the macrophage polarization experiment, the average viability of unpolarized macrophages was greater than 80%, and there were no significant differences in viability between cells processed by different methods. After 48 h of incubation with polarization medium, there was a general reduction in the viability of both the M1 and M2 macrophages but there remained no significant differences between processing cohorts. Previous studies using the THP-1 monocyte/macrophage cell line reported that prolonged (> 12–24 h) exposure to shear stress (12 dyne cm^−^^2^) within unidirectional laminar flow induces production of pro-inflammatory M1-like markers [5]. This was not observed in our experiment using flow with substantially higher shear stresses at the inlet (see dyne cm^−^^2^ in table 2), although importantly, monocytes experienced these stresses for less than 1 s within the IF device due to the high processing rate. In our experiment, relative to unpolarized macrophages, CD209 expression was greater in M2 populations than in M1 populations; however, there was no significant difference observed between these macrophage subtypes (data not shown). Electronic supplementary material, figure S2, shows optical microscope images highlighting differences in cell morphology between the classically activated M1 macrophages (electronic supplementary material, figure S2*a*) and alternatively activated M2 macrophages (electronic supplementary material, figure S2*b*). The M1-like macrophages were characterized by rounder morphology, whereas the M2-like macrophages had an elongated, rod-like morphology. These observed phenotypes are well known and are consistent with those reported in other studies [71,72]. Together, these data show no detected effects resulting from the processing of monocytes using the IF device and their subsequent ability of differentiated macrophages to undergo polarization.

#### 3.2.3. Effects of processing on cellular metabolomics

The fast-rate processing using the IF device generates high shear forces with effects that were largely unknown, but we assume fluid flow with higher Reynolds number generates higher shear stresses on cellular material. Culture medium was harvested and analysed by gas chromatography to assess if the high flow rate/Re generated from IF processing had any impact on cellular metabolism. There were no significant differences in the concentration of glucose (figure 6*a*) and lactate (figure 6*b*) between monocyte cultures processed with different methods on either day 1 or day 5. This finding was also supported by data showing no significant differences in culture pH between processing groups (figure 6*c*). The pH of the culture is altered by CO_2_ concentration and various metabolites other than lactate and therefore provides further evidence that monocyte metabolism during differentiation remains consistent independent of processing technique. Surprisingly, these data contradict the PBMC metabolomics data (figure 3*d–e*) and in monocytes there was no increase in glucose consumption at day 1 in cultures processed with the IF device, indicating other leucocyte subtypes were preferentially impacted in the PBMC.

Similarly, during macrophage polarization, there were no significant differences between processing cohorts in terms of glucose use (figure 6*d*), lactate concentration (figure 6*e*) and culture pH (figure 6*f*). This provides further evidence of a subpopulation of PBMC effecting the change in culture metabolic rate resulting from IF device processing. Together, these data indicate both monocyte and macrophage cellular metabolism are not impacted by the forces experienced during IF device processing, possibly because the channel residency time of processed cells at all flow rates was less than 0.2 s and therefore monocytes and macrophages experience these high forces for such a short period as to not have any long-term effect on their cellular metabolism. Although glucose consumption between M1 and M2 subtypes remained similar, there was a greater production of lactate in M1 macrophages compared with M2 macrophages which may be explained by a preference of M1 macrophages to metabolize glucose through glycolysis and therefore producing pyruvate rather than lactate as a metabolite [73]. This is likely due to the upregulation of the expression of glucose transporter protein, GLUT1, which increases the uptake of glucose in M1 macrophages [74]. Nevertheless, the differences in metabolic activity observed between M1 and M2 subtypes are consistent across different processing techniques and provide further evidence that the processing of monocytes with IF device does not later impact on the ability of differentiated macrophages to undergo polarization.

The adoption of IF devices within cell therapy manufacturing processes remains unrealized despite a high capacity for automation, low cost, and fully closable systems. Small changes in size or shape between phenotypically similar cells such as monocytes and macrophages [20] may require different parameters for optimal processing. Additionally, the clogging of microfluidic channels with cellular material remains a challenge [21] but some commercialized microfluidic devices have reportedly resolved this issue [22]. Before these remaining issues can be tackled it is critical to determine minimal impact on cells due to processing.

## 4. Conclusion

We present characterization of a high throughput IF device, suitable for cell separation and medium exchange, and potentially capable of integration within a closed system of cellular therapy manufacturing. The IF device separated approximately 97% of CD14+ monocytes and PBMCs to one outlet at 15 ml min^−1^ with post-processing effects on viability which were comparable to conventional techniques. Single-pass processing demonstrated lower efficiency of medium exchange compared with an alternative non-centrifugation/non-microfluidic device (Lovo, Fresenius Kabi) but did demonstrate a large reduction in potential cost and processing time, and there is the potential with this type of device to multiplex, thereby substantially increasing the processing capacity. Repeated recirculation of processed material increased the efficiency of medium exchange and was shown to be well tolerated for at least three re-circulations. Additionally, there were minimal changes to immunophenotype and metabolic activity of both monocytes and macrophages following IF processing suggesting that these functions may be unaffected. Further investigation is required to optimize the IF device design and improve medium exchange. A number of other approaches exist for processing cells in bulk at large scale including spinning membrane or counter-centrifugation devices, but all are costly to run and maintain and small, scalable IF devices offer an alternative approach. The successful development of an IF device has the potential to revolutionize this field by reducing the time, cost and complexity of cellular therapy manufacturing.

## Data Availability

Relevant data are available from the OSFHome repository: https://osf.io/h834u/ [75]. Electronic supplementary material is available online [76].

## References

[1] Emerson J, Kara B, Glassey J. 2020 Multivariate data analysis in cell gene therapy manufacturing. Biotechnol. Adv. **45**, 107637. (10.1016/j.biotechadv.2020.107637) 32980438

[2] Bellino S, La Salvia A, Cometa MF, Botta R. 2023 Cell-based medicinal products approved in the European Union: current evidence and perspectives. Front. Pharmacol. **14**, 1200808. (10.3389/fphar.2023.1200808) 37583902 PMC10424920

[3] El-Kadiry AE, Rafei M, Shammaa R. 2021 Cell therapy: types, regulation, and clinical benefits. Front. Med. (Lausanne) **8**, 756029. (10.3389/fmed.2021.756029) 34881261 PMC8645794

[4] Aranda Hernandez J, Heuer C, Bahnemann J, Szita N. 2021 Microfluidic devices as process development tools for cellular therapy manufacturing. In Microfluidics in biotechnology (eds J Bahnemann, A Grünberger), pp. 101-127. Cham, Switzerland: Springer. (10.1007/10_2021_169) 34410457

[5] Moutsatsou P, Ochs J, Schmitt RH, Hewitt CJ, Hanga MP. 2019 Automation in cell and gene therapy manufacturing: from past to future. Biotechnol. Lett. **41**, 1245-1253. (10.1007/s10529-019-02732-z) 31541330 PMC6811377

[6] Arora M. 2013 Cell culture media: a review. Mater. Methods **3**, 24. (10.13070/mm.en.3.175)

[7] Valihrach L, Androvic P, Kubista M. 2018 Platforms for single-cell collection and analysis. Int. J. Mol. Sci. **19**, 807. (10.3390/ijms19030807) 29534489 PMC5877668

[8] Lu M, Lezzar DL, Vörös E, Shevkoplyas SS. 2019 Traditional and emerging technologies for washing and volume reducing blood products. J. Blood Med. **10**, 37-46. (10.2147/JBM.S166316) 30655711 PMC6322496

[9] Li A, Kusuma GD, Driscoll D, Smith N, Wall DM, Levine BL, James D, Lim R. 2021 Advances in automated cell washing and concentration. Cytotherapy **23**, 774-786. (10.1016/j.jcyt.2021.04.003) 34052112

[10] Ibenana L et al. 2022 Assessment of the LOVO device for final harvest of novel cell therapies: a production assistance for cellular therapies multi-center study. Cytotherapy **24**, 691-698. (10.1016/j.jcyt.2022.01.010) 35279374 PMC9232931

[11] Francis N et al. 2023 Development of an automated manufacturing process for large-scale production of autologous T cell therapies. Mol. Therapy Methods Clin. Dev. **31**, 101114. (10.1016/j.omtm.2023.101114) PMC10544074 37790245

[12] Santurette CC, Charron M, Bouyer S, Houzé P, Binninger S, Lavergne A, Mercier M, Giraud C. 2022 Study of a new device for washing and concentrating cryopreserved hematopoietic stem cells and mononuclear cells: a single center experience. Cytotherapy **24**, 86-92. (10.1016/j.jcyt.2021.07.008) 34690062

[13] Delahaye M, Lawrence K, Ward SJ, Hoare M. 2015 An ultra scale-down analysis of the recovery by dead-end centrifugation of human cells for therapy. Biotechnol. Bioeng. **112**, 997-1011. (10.1002/bit.25519) 25545057 PMC4402021

[14] Acosta-Martinez J, Papantoniou I, Lawrence K, Ward S, Hoare M. 2010 Ultra scale-down stress analysis of the bioprocessing of whole human cells as a basis for cancer vaccines. Biotechnol. Bioeng. **107**, 953-963. (10.1002/bit.22888) 20677218

[15] Young EWK, Beebe DJ. 2010 Fundamentals of microfluidic cell culture in controlled microenvironments. Chem. Soc. Rev. **39**, 1036-1048. (10.1039/b909900j) 20179823 PMC2967183

[16] Halldorsson S, Lucumi E, Gómez-Sjöberg R, Fleming RMT. 2015 Advantages and challenges of microfluidic cell culture in polydimethylsiloxane devices. Biosens. Bioelectron. **63**, 218-231. (10.1016/j.bios.2014.07.029) 25105943

[17] Martel JM, Toner M. 2014 Inertial focusing in microfluidics. Annu. Rev. Biomed. Eng. **16**, 371-396. (10.1146/annurev-bioeng-121813-120704) 24905880 PMC4467210

[18] Gou Y, Jia Y, Wang P, Sun C. 2018 Progress of inertial microfluidics in principle and application. Sensors (Basel) **18**, 1762. (10.3390/s18061762) 29857563 PMC6021949

[19] Convery N, Gadegaard N. 2019 30 years of microfluidics. Micro Nano Eng. **2**, 76-91. (10.1016/j.mne.2019.01.003)

[20] Kalyan S, Torabi C, Khoo H, Sung HW, Choi SE, Wang W, Treutler B, Kim D, Hur SC. 2021 Inertial microfluidics enabling clinical research. Micromachines (Basel) **12**, 257. (10.3390/mi12030257) 33802356 PMC7999476

[21] Segre G, Silberberg A. 1961 Radial particle displacements in Poiseuille flow of suspensions. Nature **189**, 209-210. (10.1038/189209a0)

[22] Di Carlo D, Irimia D, Tompkins RG, Toner M. 2007 Continuous inertial focusing, ordering, and separation of particles in microchannels. Proc. Natl Acad. Sci. USA **104**, 18892-18897. (10.1073/pnas.0704958104) 18025477 PMC2141878

[23] Guzniczak E, Otto O, Whyte G, Willoughby N, Jimenez M, Bridle H. 2020 Deformability-induced lift force in spiral microchannels for cell separation. Lab Chip **20**, 614-625. (10.1039/C9LC01000A) 31915780

[24] Dean WR. 1928 LXXII. The stream-line motion of fluid in a curved pipe (second paper). Lond. Edinb. Dublin Phil. Mag. J. Sci. **5**, 673-695. (10.1080/14786440408564513)

[25] Ramachandraiah H, Ardabili S, Faridi AM, Gantelius J, Kowalewski JM, Mårtensson G, Russom A. 2014 Dean flow-coupled inertial focusing in curved channels. Biomicrofluidics **8**, 034117. (10.1063/1.4884306) 25379077 PMC4162445

[26] Lee J-H, Lee S-K, Kim J-H, Park J-H. 2019 Separation of particles with bacterial size range using the control of sheath flow ratio in spiral microfluidic channel. Sens. Actuators A: Phys. **286**, 211-219. (10.1016/j.sna.2018.12.047)

[27] Moloudi R, Oh S, Yang C, Ebrahimi Warkiani M, Naing MW. 2018 Inertial particle focusing dynamics in a trapezoidal straight microchannel: application to particle filtration. Microfluid Nanofluid **22**, 33. (10.1007/s10404-018-2045-5)

[28] Wang L, Dandy DS. 2017 High-throughput inertial focusing of micrometer- and sub-micrometer-sized particles separation. Adv. Sci. **4**, 1700153. (10.1002/advs.201700153) PMC5644225 29051857

[29] Zhang J, Yan S, Sluyter R, Li W, Alici G, Nguyen N-T. 2014 Inertial particle separation by differential equilibrium positions in a symmetrical serpentine micro-channel. Sci. Rep. **4**, 4527. (10.1038/srep04527) 24681628 PMC3970124

[30] Zhang J, Yan S, Li W, Alici G, Nguyen N-T. 2014 High throughput extraction of plasma using a secondary flow-aided inertial microfluidic device. RSC Adv. **4**, 33 149-33 159. (10.1039/C4RA06513A)

[31] Kim J-A, Kommajosula A, Choi Y-H, Lee J-R, Jeon E-C, Ganapathysubramanian B, Lee W. 2020 Inertial focusing in triangular microchannels with various apex angles. Biomicrofluidics **14**, 024105. (10.1063/1.5133640) 32231759 PMC7093208

[32] Garcia-Briones MA, Chalmers JJ. 1994 Flow parameters associated with hydrodynamic cell injury. Biotechnol. Bioeng. **44**, 1089-1098. (10.1002/bit.260440910) 18623026

[33] Warkiani ME et al. 2014 Slanted spiral microfluidics for the ultra-fast, label-free isolation of circulating tumor cells. Lab Chip **14**, 128-137. (10.1039/C3LC50617G) 23949794

[34] Kwon T, Prentice H, Oliveira JD, Madziva N, Warkiani ME, Hamel J-FP, Han J. 2017 Microfluidic cell retention device for perfusion of mammalian suspension culture. Sci. Rep. **7**, 6703. (10.1038/s41598-017-06949-8) 28751635 PMC5532224

[35] Zhu S, Wu D, Han Y, Wang C, Xiang N, Ni Z. 2020 Inertial microfluidic cube for automatic and fast extraction of white blood cells from whole blood. Lab Chip **20**, 244-252. (10.1039/C9LC00942F) 31833515

[36] Ramachandraiah H, Svahn HA, Russom A. 2017 Inertial microfluidics combined with selective cell lysis for high throughput separation of nucleated cells from whole blood. RSC Adv. **7**, 29 505-29 514. (10.1039/C7RA02992F)

[37] Armistead FJ, Gala De Pablo J, Gadêlha H, Peyman SA, Evans SD. 2019 Cells under stress: an inertial-shear microfluidic determination of cell behavior. Biophys. J. **116**, 1127-1135. (10.1016/j.bpj.2019.01.034) 30799072 PMC6428867

[38] Connolly S, McGourty K, Newport D. 2020 The *in vitro* inertial positions and viability of cells in suspension under different *in vivo* flow conditions. Sci. Rep. **10**, 1711. (10.1038/s41598-020-58161-w) 32015362 PMC6997401

[39] Xiang N, Ni Z. 2015 High-throughput blood cell focusing and plasma isolation using spiral inertial microfluidic devices. Biomed. Microdevices **17**, 110. (10.1007/s10544-015-0018-y) 26553099

[40] Liu Y, Zhao W, Cheng R, Puig A, Hodgson J, Egan M, Cooper Pope CN, Nikolinakos PG, Mao L. 2021 Label-free inertial-ferrohydrodynamic cell separation with high throughput and resolution. Lab Chip **21**, 2738-2750. (10.1039/D1LC00282A) 34018527

[41] Dudani JS, Gossett DR, Tse HTK, Lamm RJ, Kulkarni RP, Carlo DD. 2015 Rapid inertial solution exchange for enrichment and flow cytometric detection of microvesicles. Biomicrofluidics **9**, 014112. (10.1063/1.4907807) 25713694 PMC4320146

[42] Gossett DR, Tse HTK, Dudani JS, Goda K, Woods TA, Graves SW, Di Carlo D. 2012 Inertial manipulation and transfer of microparticles across laminar fluid streams. Small **8**, 2757-2764. (10.1002/smll.201200588) 22761059

[43] Mfarrej B et al. 2017 Pre-clinical assessment of the Lovo device for dimethyl sulfoxide removal and cell concentration in thawed hematopoietic progenitor cell grafts. Cytotherapy **19**, 1501-1508. (10.1016/j.jcyt.2017.09.001) 29037941

[44] Bhagat AA, Kuntaegowdanahalli S, Papautsky I. 2008 Inertial microfluidics for continuous particle filtration and extraction. Microfluid. Nanofluid. **7**, 217-226. (10.1007/s10404-008-0377-2)

[45] Xiang N, Shi Z, Tang W, Huang D, Zhang X, Ni Z. 2015 Improved understanding of particle migration modes in spiral inertial microfluidic devices. RSC Adv. **5**, 77 264-77 273. (10.1039/C5RA13292D)

[46] Martel JM, Toner M. 2012 Inertial focusing dynamics in spiral microchannels. Phys. Fluids **24**, 032001. (10.1063/1.3681228) PMC3311666 22454556

[47] Fraser AR et al. 2017 Development, functional characterization and validation of methodology for GMP-compliant manufacture of phagocytic macrophages: a novel cellular therapeutic for liver cirrhosis. Cytotherapy **19**, 1113-1124. (10.1016/j.jcyt.2017.05.009) 28673774 PMC5571439

[48] Huang D, Man J, Jiang D, Zhao J, Xiang N. 2020 Inertial microfluidics: recent advances. Electrophoresis **41**, 2166-2187. (10.1002/elps.202000134) 33027533

[49] Karabacak NM et al. 2014 Microfluidic, marker-free isolation of circulating tumor cells from blood samples. Nat. Protoc. **9**, 694-710. (10.1038/nprot.2014.044) 24577360 PMC4179254

[50] Bakhshi MS, Rizwan M, Khan GJ, Duan H, Zhai K. 2022 Design of a novel integrated microfluidic chip for continuous separation of circulating tumor cells from peripheral blood cells. Sci. Rep. **12**, 17016. (10.1038/s41598-022-20886-1) 36220844 PMC9554048

[51] Fukuda S, Schmid-Schönbein GW. 2002 Centrifugation attenuates the fluid shear response of circulating leukocytes. J. Leukoc. Biol. **72**, 133-139. (10.1189/jlb.72.1.133) 12101272

[52] Aleksandrova K et al. 2019 Functionality and cell senescence of CD4/ CD8-selected CD20 CAR T cells manufactured using the automated CliniMACS Prodigy® platform. Transfus. Med. Hemother. **46**, 47-54. (10.1159/000495772) 31244581 PMC6558326

[53] Zhou Y, Ma Z, Ai Y. 2018 Sheathless inertial cell focusing and sorting with serial reverse wavy channel structures. Microsyst. Nanoeng. **4**, 5. (10.1038/s41378-018-0005-6) 31057895 PMC6220157

[54] Moroni F et al. 2019 Safety profile of autologous macrophage therapy for liver cirrhosis. Nat. Med. **25**, 1560-1565. (10.1038/s41591-019-0599-8) 31591593

[55] Cheung M, Campbell JJ, Thomas RJ, Braybrook J, Petzing J. 2022 Assessment of automated flow cytometry data analysis tools within cell and gene therapy manufacturing. Int. J. Mol. Sci. **23**, 3224. (10.3390/ijms23063224) 35328645 PMC8955358

[56] Kapellos TS, Bonaguro L, Gemünd I, Reusch N, Saglam A, Hinkley ER, Schultze JL. 2019 Human monocyte subsets and phenotypes in major chronic inflammatory diseases. Front. Immunol. **10**, 2035. (10.3389/fimmu.2019.02035) 31543877 PMC6728754

[57] Zamani F, Zare Shahneh F, Aghebati-Maleki L, Baradaran B. 2013 Induction of CD14 expression and differentiation to monocytes or mature macrophages in promyelocytic cell lines: new approach. Adv. Pharm. Bull. **3**, 329-332. (10.5681/apb.2013.053) 24312856 PMC3848216

[58] Wang H, Riha GM, Yan S, Li M, Chai H, Yang H, Yao Q, Chen C. 2005 Shear stress induces endothelial differentiation from a murine embryonic mesenchymal progenitor cell line. Arterioscler. Thromb. Vasc. Biol. **25**, 1817-1823. (10.1161/01.ATV.0000175840.90510.a8) 15994439

[59] Wong KL, Tai JJ-Y, Wong WC, Han H, Sem X, Yeap W-H, Kourilsky P, Wong S-C. 2011 Gene expression profiling reveals the defining features of the classical, intermediate, and nonclassical human monocyte subsets. Blood **118**, e16-e31. (10.1182/blood-2010-12-326355) 21653326

[60] Bayik D, Tross D, Haile LA, Verthelyi D, Klinman DM. 2017 Regulation of the maturation of human monocytes into immunosuppressive macrophages. Blood Adv. **1**, 2510-2519. (10.1182/bloodadvances.2017011221) 29296902 PMC5728638

[61] Relloso M et al. 2002 DC-SIGN (CD209) expression is IL-4 dependent and is negatively regulated by IFN, TGF-β, and anti-inflammatory agents. J. Immunol. **168**, 2634-2643. (10.4049/jimmunol.168.6.2634) 11884427

[62] Barth E, Fischer G, Schneider EM, Wollmeyer J, Georgieff M, Weiss M. 2001 Differences in the expression of CD64 and mCD14 on polymorphonuclear cells and on monocytes in patients with septic shock. Cytokine **14**, 299-302. (10.1006/cyto.2001.0880) 11444911

[63] Serbina NV, Pamer EG. 2006 Monocyte emigration from bone marrow during bacterial infection requires signals mediated by chemokine receptor CCR2. Nat. Immunol. **7**, 311-317. (10.1038/ni1309) 16462739

[64] Zhao BN et al. 2019 CCR2-mediated uptake of constitutively produced CCL2: a mechanism for regulating chemokine levels in the blood. J. Immunol. **203**, 3157-3165. (10.4049/jimmunol.1900961) 31676674 PMC7028331

[65] Sica A, Erreni M, Allavena P, Porta C. 2015 Macrophage polarization in pathology. Cell. Mol. Life Sci. **72**, 4111-4126. (10.1007/s00018-015-1995-y) 26210152 PMC11113543

[66] Boutilier AJ, Elsawa SF. 2021 Macrophage polarization states in the tumor microenvironment. Int. J. Mol. Sci. **22**, 6995. (10.3390/ijms22136995) 34209703 PMC8268869

[67] Wang C, Ma C, Gong L, Guo Y, Fu K, Zhang Y, Zhou H, Li Y. 2021 Macrophage polarization and its role in liver disease. Front. Immunol. **12**, 803037. (10.3389/fimmu.2021.803037) 34970275 PMC8712501

[68] Funes SC, Rios M, Escobar-Vera J, Kalergis AM. 2018 Implications of macrophage polarization in autoimmunity. Immunology **154**, 186-195. (10.1111/imm.12910) 29455468 PMC5980179

[69] Ngambenjawong C, Gustafson HH, Pun SH. 2017 Progress in tumor-associated macrophage (TAM)-targeted therapeutics. Adv. Drug Deliv. Rev. **114**, 206-221. (10.1016/j.addr.2017.04.010) 28449873 PMC5581987

[70] Gauthier T, Chen W. 2022 Modulation of macrophage immunometabolism: a new approach to fight infections. Front. Immunol. **13**, 780839. (10.3389/fimmu.2022.780839) 35154105 PMC8825490

[71] McWhorter FY, Wang T, Nguyen P, Chung T, Liu WF. 2013 Modulation of macrophage phenotype by cell shape. Proc. Natl Acad. Sci. USA **110**, 17 253-17 258. (10.1073/pnas.1308887110) PMC3808615 24101477

[72] Heinrich F et al. 2017 Morphologic, phenotypic, and transcriptomic characterization of classically and alternatively activated canine blood-derived macrophages *in vitro*. PLoS ONE **12**, e0183572. (10.1371/journal.pone.0183572) 28817687 PMC5560737

[73] Russo S, Kwiatkowski M, Govorukhina N, Bischoff R, Melgert BN. 2021 Meta-inflammation and metabolic reprogramming of macrophages in diabetes and obesity: the importance of metabolites. Front. Immunol. **12**, 746151. (10.3389/fimmu.2021.746151) 34804028 PMC8602812

[74] Viola A, Munari F, Sánchez-Rodríguez R, Scolaro T, Castegna A. 2019 The metabolic signature of macrophage responses. Front. Immunol. **10**, 1462. (10.3389/fimmu.2019.01462) 31333642 PMC6618143

[75] Carvell T, Burgoyne P, Milne L, Campbell JDM, Fraser AR, Bridle H. 2024 Data from: Human leucocytes processed by fast-rate inertial microfluidics retain conventional functional characteristics. OSFHome repository. (https://osf.io/h834u/)10.1098/rsif.2023.057238442860

[76] Carvell T, Burgoyne P, Milne L, Campbell JDM, Fraser AR, Bridle H. 2024 Human leucocytes processed by fast-rate inertial microfluidics retain conventional functional characteristics. Figshare. (10.6084/m9.figshare.c.7099722)38442860

